# Maternal consumption of fish oil protected breast-fed piglets against *Escherichia coli* lipopolysaccharide-induced damage through reshaping of intestinal fatty acids profile

**DOI:** 10.3389/fvets.2024.1417078

**Published:** 2024-06-17

**Authors:** Bo Fang, Lianpeng Zhao, Bin Huo, Fangyuan Chen, Peiqiang Yuan, Shanshan Lai, Aimin Wu, Yong Zhuo

**Affiliations:** Key Laboratory for Animal Disease Resistance Nutrition of the Ministry of Education, Animal Nutrition Institute, Sichuan Agricultural University, Chengdu, China

**Keywords:** sow, fish oil, lipopolysaccharide, intestine, inflammation

## Abstract

It has been well documented that n-3 polyunsaturated fatty acids (n-3 PUFA) can alleviate inflammation caused by *Escherichia coli* (*E. coli*) lipopolysaccharides (LPS), the etiologic agents that causing yellow or white dysentery in young pigs. However, it remains unclear whether the increase in n-3 PUFA availability could enhance the ability of nursery pigs to resist invasion by *E. coli*. LPS. Twenty-four 21-day-old female piglets, each two of them from the same sow fed the beef tallow (BT) or fish oil (FO) diets, were allocated into four treatment groups: BT-CON, piglets from the BT-fed sows and intraperitoneally injected with saline (9 g/L); BT-LPS, piglets from the BT-fed sows and injected with LPS (100 μg/kg body weight); FO-CON, piglets from the FO-fed sows and injected with saline; FO-LPS, piglets from the FO-fed sows and injected with LPS. Following 2 h of LPS challenge, the magnitudes of increase in body temperature approached to a marked (*p <* 0.01) difference between the BT-CON and BT-LPS piglets, whereas the dramatic (*p <* 0.01) difference between the FO-CON and FO-LPS piglets was only observed at 4 h post LPS challenge. The body temperature averaged across the time points evaluated was about 0.2°C lower (*p <* 0.05) in the FO group than in the BT group. The FO group had lower (*p <* 0.05) mean corpuscular hemoglobin concentration, lower increase in serum interleukin (IL)-1β (*p* < 0.10) and IL-8 (*p <* 0.05) levels, higher (*p <* 0.01) serum albumin concentration, and higher (*p* = 0.10) ratios of jejunum villus height to crypt depth than the BT group. The FO group had much higher (*p <* 0.0001) ileal content of C20:5n3, C24:0, and C22:6n3, which were 2–4 times the content of the BT group. LPS challenge resulted in decreased (*p <* 0.05) intestinal C20:1 and C20:5n3 content, and the decrease (*p <* 0.05) in intestinal C20:3n6 and C24:1 content was observed in the BT-LPS piglets rather than in the FO-LPS piglets. Taken together, this study indicated that maternal consumption of fish oil protected breast-fed piglets against *E. coli* LPS-induced damage through reshaping of intestinal fatty acids profile, which sheds new light on the development of nutritional strategies to enhance the ability of young pigs to resist *E. coli* invasion.

## Introduction

Pathogenic *Escherichia coli* (*E. coli*) has long been known to be one of the major etiologic agents threatening the health of pigs. Infection of pigs by *E. coli* typically results in diarrhea, enteritis or enterotoxemia (1–3). This seriously hinders pig growth and decreases feed utilization efficiency. It is well documented that the inflammatory diseases caused by *E. coli* are mainly ascribed to the presence of lipopolysaccharides (LPS) in its outer cell membrane (4–6). The cells of the immune system are known to rely on the pattern recognition receptor Toll-like receptor 4 (TLR4) to recognize extracellular LPS (7–9). TLR4, once bound to its ligand LPS, activates nuclear factor-κB (NF-κB), which subsequently causes the secretion of pro-inflammatory cytokines such as interleukin-1β (IL-1β), IL-6 and tumor necrosis factor-alpha (TNF-α) (10–13), leading to a series of inflammatory reactions to resist LPS infection (14–16).

Although the inflammatory responses are considered necessary for cells to resist infection by pathogens, the excessive inflammatory response is also harmful to the organism. Growing evidence supports that limiting the intensity of TLR signal response is an effective way to control harmful inflammatory diseases (17–21). Numerous *in vitro* studies have confirmed that n-3 polyunsaturated fatty acids (n-3 PUFA), especially eicosapentaenoic acid (EPA) and docosahexaenoic acid (DHA) can inhibit the activation of TLR4 signaling pathway by SFA such as lauric acid and palmitic acid and LPS at the receptor level (22–26). These results imply the potential role of dietary fat type in regulating animal’s resistance to pathogen infection and inflammatory response.

Given that *E. coli* is the etiologic agent that causing yellow or white dysentery, which seriously threatens the intestinal health of nursery pigs (27), there is growing interest in exploring the nutritional strategies to deal with the challenge by *E. coli* (28–33). A previous study in rats fed fish oil diet has indicated the downregulation of the pro-inflammatory cytokine IL-1β and the upregulation of the anti-inflammatory factor IL-10 following LPS challenge in the early stage of delivery (34). Further research in lactating sows fed fish oil indicated the improved weaning survival rate of piglets, the increase of immunoglobulin (Ig) G and IgM levels in colostrum and milk (35), and the increase of EPA and DHA levels in breast milk and serum of piglets, showing a significant role of dietary fat in regulating fatty acid composition of breast milk and piglets. However, it remains unclear whether the increase in n-3 PUFA availability could enhance the ability of piglets to resist invasion by *E. coli*. LPS.

Considering the goalkeeper role of the enterocytes in resisting invasion by pathogens, and that PUFAs are structural components of membrane phospholipids, and influence cellular function via effects on membrane properties, and also by acting as a precursor pool for lipid mediators (36), we hypothesized that maternal consumption of diets rich in n-3 PUFA could enhance the ability of piglets to resist LPS invasion by modulating fatty acid profile of the intestine. To test this hypothesis, *E. coli*. LPS was used as the stressor to challenge the breast-fed piglets from the sows fed diets different in fatty acid composition. Body temperature and blood biochemical indexes were determined to evaluate the LPS induced injury, and intestinal morphology combined with fatty acid profile were determined to reveal the potential protection pathways of maternal n-3 PUFA consumption on their progeny.

## Materials and methods

### Ethics approval

The guidelines of this study were approved by the Animal Care and Use Committee of the Animal Nutrition Institute (2022092114008), Sichuan Agricultural University. The study was conducted following the guidelines of the National Research Council’s Guide for the Care and Use of Laboratory Animals, Chinese Order No. 676 of the State Council (1 March 2017).

### Experimental design and animal feeding management

Twenty crossbred [Duroc × (Landrace × York)] replacement gilts, artificially inseminated following synchronization of estrus, were fed the same diet from the day of insemination (gestation day 0) to gestation day 106. On day 107 of gestation, sows were moved into farrowing crates (2.1 × 0.7 m) with an area (2.1 × 0.6 m) allocated to newborn piglets on each side of the crates in an environmentally regulated farrowing house. From gestation day 107 until weaning, sows were fed one of the two experimental diets: beef tallow (BT) diet (basal diet +4.6% of BT), fish oil (FO) diet (basal diet +4.6% of FO), with 10 sows per treatment. The feed allowance for each treatment of sows was 2.5 kg/day at the first 4 days, followed by 2 kg/day, and no feed was provided at the farrowing day. Two kilograms of feed was provided at postpartum day 1, and then increased by 1 kg/day until postpartum day 7, after which feed was offered *ad libitum*. The diets were formulated based on NRC (2012) recommendation (see Table 1). The BT product (purity 99.93%) was purchased from Sichuan Yizhou Grease Co., Ltd. (Chengdu, China). The FO product was purchased from Xi’an Xihai Bio-technique Co., Ltd. (Xi’an, China), containing 30% of EPA (C20:5n3) and DHA (C22:6n3). The grease products were stored at −18°C refrigerator until using. The liquid BT was obtained by heating upon using. The required amounts of oils were directly and uniformly poured into the mixer from the alternative feed inlet of the mixer at a constant speed. Considering that it’s a common practice that oils were included in the commercial feed of lactating sows, the diet without oil supplementation were not used as the control. On the 21st day of lactation, 6 sows with similar weight and litter size were selected from each of the treatment for the selection of experimental piglets. Two healthy female piglets with similar body weight were selected from each sow and randomly assigned to the LPS challenge group and control (CON) group, respectively. Six of piglets in each treatment were intraperitoneally injected with LPS solution to administer LPS at 100 μg/kg BW to make a model of immunological challenge, and another 6 piglets in each treatment were intraperitoneally injected with an equivalent amount of sterile saline. As a result, four treatment groups were formed: BT-CON (piglets selected from the BT-fed sows and injected with saline), BT-LPS (piglets selected from the BT-fed sows and injected with LPS solution), FO-CON (piglets selected from the FO-fed sows and injected with saline), and FO-LPS (piglets selected from the FO-fed sows and injected with LPS solution). Piglets were separated from sows 4 h before the injections. The LPS (Sigma-Aldrich, Los Angeles, California) was dissolved in sterile saline (9 g/L) to make LPS solution (1,000 mg LPS/L saline). Dosage of LPS injection were determined by a pretest conducted using the piglets that were not included in the formal testing. The time to sacrifice piglets were previously described.

**Table 1:** Ingredients and composition of in the experimental diets fed to lactation sows.

	BT diet	FO diet
**Raw material composition, %**		
Wheat	20.00	20.00
Corn	43.90	43.90
Soybean meal	7.50	7.50
Rapeseed meal	19.10	19.10
Beef tallow (BT)	4.60	0.00
Fish oil (FO)	0.00	4.60
DL-methionine hydroxy analog	0.10	0.10
L-lysine	0.40	0.40
L-threonine	0.10	0.10
Limestone	0.20	0.20
Calcium hydrogen phosphate	2.20	2.20
Sodium chloride	0.40	0.40
Premix^a^^,^^b^^,^^c^	1.50	1.50
**Chemical composition, %**		
Protein	16.20	16.20
Starch	35.70	35.70
Fat	7.00	7.00
Net energy, MJ/kg	10.53	10.53
**Digestible amino acids, %**		
Arginine	0.82	0.82
Histidine	0.36	0.36
Isoleucine	0.53	0.53
Leucine	1.12	1.12
Lysine	0.92	0.92
Methionine	0.31	0.31
Methionine + cysteine	0.48	0.48
Phenylalanine	0.61	0.61
Phenylalanine + tyrosine	1.06	1.06
Threonine	0.58	0.58
Tryptophan	0.18	0.18
Valine	0.63	0.63
**Minerals, %**		
Calcium	0.90	0.90
Digestible phosphorus	0.50	0.50

a Provided per kg of diet: vitamin A, 6000 IU; vitamin D_3_, 1,200 IU; vitamin E, 50 IU; menadione, 2.40 mg; thiamine, 1 mg; riboflavin, 3.60 mg; niacin, 20 mg; d-panthothenic acid, 12.50 mg; vitamin B_6_, 1.80 mg; vitamin B_12_, 12.50 μg; d-biotin, 240 μg; folic acid, 2 mg.

b Provided per kg of diet: copper, 25 mg; iron, 100 mg; manganese, 35 mg; zinc, 125 mg; iodine, 0.20 mg; selenium, 0.30 mg.

c Provided per kg of diet: anti-mould (sodium propionate), 0.50 g; mycotoxin adsorbent (montmorillonoid), 1 g; anti-oxidants (ethoxyquin & propionate gallate), 0.20 g.

### Sample collection

Piglets were immediately transferred to metabolic cages after injection of LPS or saline for 4 h of observation. During the experiment, the conditions of piglets were closely observed and clinical symptoms such as diarrhea, vomiting, and skin cyanosis were recorded. At 0, 2 and 4 h after injection of LPS or normal saline, the rectal temperature of each piglet was measured. Blood samples were collected at 0 and 4 h from the anterior vena cava into heparinized tubes (~10 mL), stood for 30 min in the ice, and then centrifuged (2,550 × g, 4°C, 10 min) and stored at −20°C until analysis. Another tube (~1.5 mL) of blood sample, added with EDTA to prevent coagulation, was kept on ice and immediately sent to lab for blood cell counts. Immediately after collection of blood samples at 4 h, pigs were euthanized by electrical stunning and exsanguination. Immediately after death, the abdominal cavity was opened, and ileal tissue samples were quickly collected as previously described (37), snap-frozen in liquid nitrogen, and stored at −80°C until analysis. Two cm-long segments of jejunum were sampled as previously described and immediately fixed in phosphate-buffered paraformaldehyde (4%, pH 7.6) for histological measurements.

### Intestinal morphology analysis

Intestinal segments were taken out from fixative solution and then dehydrated with increasing concentrations of ethanol and chloroform. The segments were processed with paraffin, and two transverse tissue samples were cut from each segment using a microtome. These parts of the tissue samples were dehydrated, embedded together in paraffin wax, and sectioned at 5 μm. One transverse tissue sample of each segment was transferred to a slide and stained with haematoxylin and eosin. Villi height (VH) and crypt depth (CD) were determined as we described previously (38). VH was measured from the tip of the villi to the base between individual villi, and CD measurements were taken from the valley between individual villi to the basal membrane.

### Biochemical analysis

Routine analysis of blood samples in EDTA venipuncture vials were performed by XE-2100 automatic blood cell analyzer (Sysmex Corporation, Kobe, Japan). The contents detected include leucocyte (neutrophil, lymphocyte and monocyte counts), and platelet.

Serum IL-1β and IL-8 concentrations were measured using the ELISA kits suitable for porcine IL-1β (Meimian industrial Co., Ltd., Jiangsu, China) and IL-8 (Nanjing JianCheng Bioengineering Institute, Inc., Nanjing, China), according to the manufacture’s protocol. The levels of IL-1β and IL-8 were calculated from the standard curve and were expressed as nanograms per liter.

Serum albumin was measured by the International Federation of Clinical Chemistry (IFCC) dynamic method using an automatic biochemical analyzer (HITACHI 3100, Tokyo, Japan) according to the instructions of kits (Nanjing Jiancheng Bioengineering Institute, Nanjing, China).

The analysis of fatty acid composition of diets, milk and piglet ileal tissue samples were performed according to the described method (32) with a minor modification. A mixed fatty acids standard (Sigma, St Louis, MO, United States) was added as an external standard. After pretreatment including hydrolysis, extraction, and saponification of fats and methylation of fatty acids, the fatty acid methyl esters were analyzed by gas chromatography–mass spectrometry (Thermo, Trace1310 ISQ) installed with a column (TG-FAME; 50 m × 0.25 mm × 0.20 μm). The chromatography conditions were as follows: 260°C injector temperature; 240°C detector temperature; and carrier gas at 1/100 split ratio: The temperature program was set to run for 80°C for 1 min, followed by an increase of 20°C/min to 160°C which maintained for 1.5 min, and followed by an increase of 6°C/min to 240°C which maintained for 6 min. The mass spectrometry conditions were as follows. 280°C ion source temperature; 240°C transmission line temperature; 5 min solvent delay time; EI source 70 eV ion source. Peaks were identified by comparison of retention times with standards. Identification of the peaks included 37 species of fatty acids between C4:0 and C24:1. Fatty acid content of samples were calculated as follows:


$$W=\frac{C\times V\times N}{m}\times k\times 10^{-3}$$

where *W* is the fatty acid content (g/kg), *C* is the fatty acid methyl esters concentration (mg/L), *V* is the fixed volume (mL), *k* is the conversion coefficients for converting methyl esters of various fatty acids into fatty acids, *N* is the dilution times, 10^−3^ is the unit conversion factor, and *m* is the weight of samples (g).

### Statistical analysis

The data were analyzed using the SAS 9.4 statistical package (SAS Inst. Inc., Cary, NC). The Shapiro–Wilk test was conducted to assess the normality of the data. Unless otherwise specified, data were analyzed by using two-way ANOVA procedures. As described by Littell et al. (39), the repeated-measures data including body temperature, blood cell counts, and serum cytokines were analyzed by using the MIXED procedure according to the following model:


$$Y_{ijk}=\mu +a_{i}+\beta_{j}+r_{k}+{\left(\alpha \beta \right)}_{ij}+{\left(\alpha \gamma \right)}_{ik}+{\left(\beta \gamma \right)}_{jk}+{\left(\alpha \beta \gamma \right)}_{ijk}+\varepsilon_{ijk}$$

where *Y* is the analyzed variable, *μ* is the mean, *α_i_* is the effect of diet (*i* = 1, 2), *β_j_* is the effect of LPS (*j* = 1, 2), *γ_k_* is the effect of time (*k* = 1, 2 or 3), (*αβ*)*_ij_* refers to the interaction between diet and LPS, (*αγ*)*_ik_* refers to the interaction between diet and time, (*βγ*)*_jk_* refers to the interaction between LPS and time, (*αβγ*)*_ijk_* refers to the interaction among diet, LPS and time, and *ε_ijk_* represents the residual error. A two-tailed unpaired *t*-test was used to determine the significance of the comparisons of data indicating the change of body temperature. Outlier data points were examined using the Grubbs’ test and removed. All differences were considered to be statistically significant at *p* < 0.05, and it was considered to have a tendency towards difference at *p* < 0.10.

## Results

### Increased n-3 PUFA content in milk of the FO-fed sows

Table 2 shows the effect of maternal consumption of BT or FO on milk fatty acid composition. A total of 22 fatty acid species were detected in the sow milk. FO consumption decreased milk C16:0 (*p <* 0.10), C18:0 (*p <* 0.05) and total SFA (*p <* 0.10) concentrations, while increased concentration of C24:0 which were not detected in the milk of the BT-fed sows. In addition to the lower (*p <* 0.05) milk C16:1 concentration, milk C18:1n9c and total MUFA concentrations in the FO group were decreased (*p <* 0.0001) to be about 45 and 55% of the contents in the BT group. Milk C20:1 (*p <* 0.0001), C22:1n9 (*p <* 0.0001), and C24:1 (*p <* 0.001) concentrations were increased to be 2–6 times that in the milk of the BT-fed sows. C18:2n6c, the predominant n-6 PUFA in the milk of the BT-fed sows was about twice that of the FO-fed sows. C20:3n3 were lower (*p <* 0.05) in the BT-fed sows than in the FO-fed sows, and, moreover, C20:5n3 and C22:6n3 were not detected in the BT-fed sows. As a result, the total n-3 PUFA in the milk of the FO-fed sows was beyond 5 times that of the BT-fed sows.

**Table 2:** Effect of maternal diets on milk fatty acid composition (g/kg milk).^a^

	Diet		*p*-value
	BT	FO	
**SFA**			
C10:0	0.10 ± 0.03	0.09 ± 0.02	NS
C12:0	0.17 ± 0.04	0.15 ± 0.03	NS
C14:0	2.62 ± 0.52	2.33 ± 0.35	NS
C15:0	0.08 ± 0.02	0.07 ± 0.01	NS
C16:0	23.07 ± 4.32	18.73 ± 2.35	^#^
C17:0	0.16 ± 0.04	0.13 ± 0.02	NS
C18:0	3.40 ± 0.91	2.15 ± 0.51	^*^
C24:0	—	0.79 ± 0.08	^****^
∑SFA	29.59 ± 5.56	24.42 ± 3.10	^#^
**MUFA**			
C14:1	0.15 ± 0.04	0.14 ± 0.02	NS
C16:1	6.79 ± 0.95	5.63 ± 0.34	^*^
C18:1n9c	22.16 ± 3.96	9.91 ± 1.46	^****^
C20:1	0.20 ± 0.06	0.41 ± 0.05	^****^
C22:1n9	0.08 ± 0.01	0.16 ± 0.01	^****^
C24:1	0.01 ± 0.02	0.06 ± 0.01	^***^
∑MUFA	29.39 ± 3.86	16.31 ± 1.54	^****^
**n-6 PUFA**			
C18:2n6c	9.01 ± 1.73	5.39 ± 0.68	^***^
C20:2	0.18 ± 0.07	0.14 ± 0.02	NS
C20:3n6	0.04 ± 0.02	0.03 ± 0.02	NS
C20:4n6	0.16 ± 0.03	0.18 ± 0.02	NS
∑n-6 PUFA	9.39 ± 1.83	5.75 ± 0.72	^**^
**n-3 PUFA**			
C18:3n3	0.32 ± 0.09	0.37 ± 0.05	NS
C20:3n3	0.05 ± 0.01	0.07 ± 0.01	^*^
C20:5n3	—	0.18 ± 0.02	^****^
C22:6n3	—	1.32 ± 0.15	^****^
∑n-3 PUFA	0.38 ± 0.10	1.95 ± 0.21	^****^
Total fatty acids	68.74 ± 9.50	48.42 ± 4.61	^***^

a SFA, saturated fatty acids; MUFA, monounsaturated fatty acids; PUFA, polyunsaturated fatty acids; —, not detected. ^*^p < 0.05, ^**^p < 0.01, ^***^p < 0.001, and ^****^p < 0.0001; NS, *p* > 0.10.

### Delayed increase of body temperature in piglets from the FO-fed sows following LPS challenge

**Figure 1:**
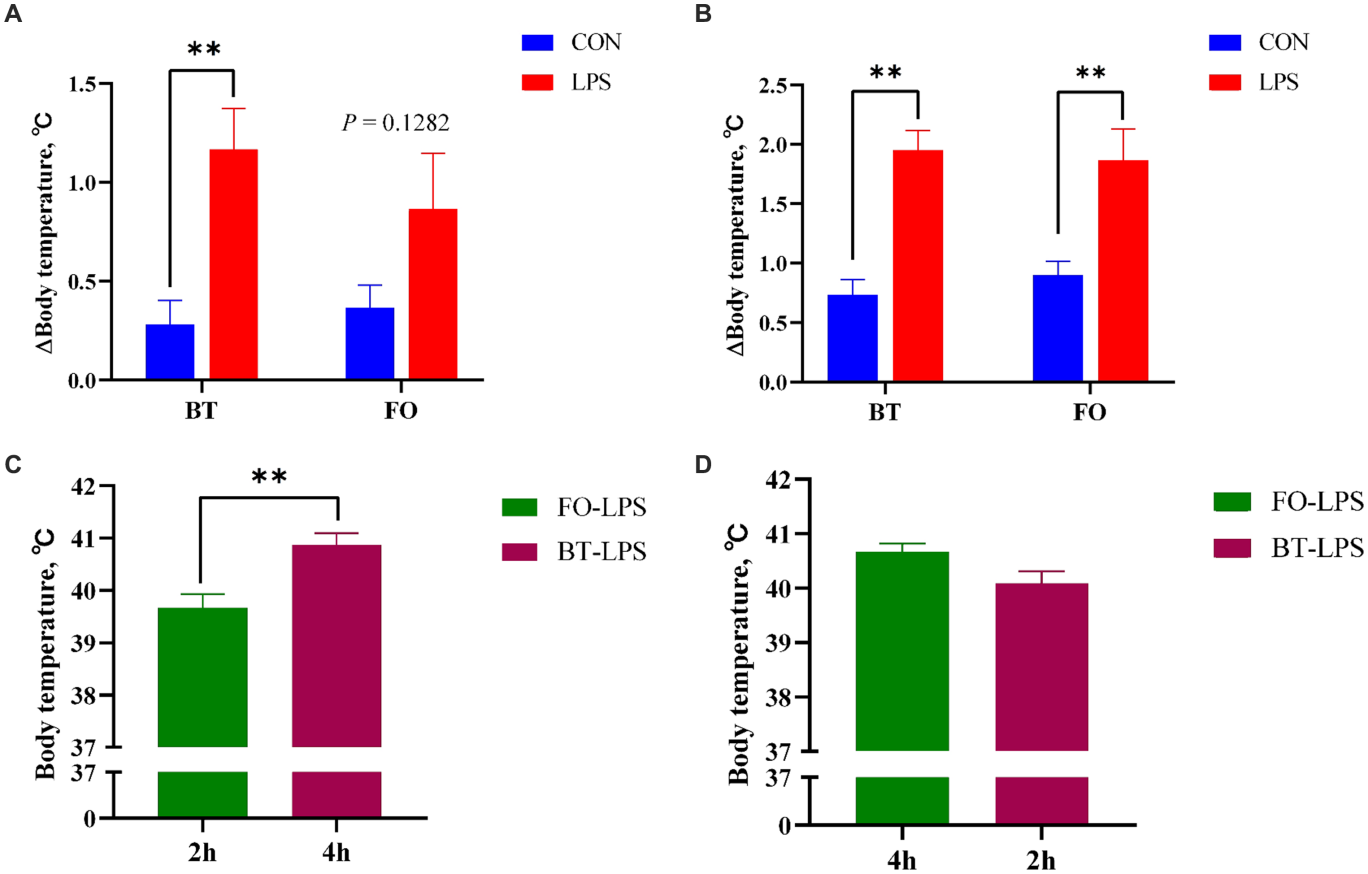
Effect of maternal consumption of beef tallow (BT) or fish oil (FO) on the change of body temperature of piglets challenged with intraperitoneal injection of *E. coli* lipopolysaccharide (LPS). **(A)** Comparison of the magnitudes of increase in body temperature following 2 h of LPS challenge between the CON and LPS-treated piglets. **(B)** Comparison of the magnitudes of increase in body temperature following 4 h of LPS challenge between the CON- and LPS-treated piglets. **(C)** Comparison of body temperature between LPS-treated piglets following 4 h (BT group) or 2 h (FO group) of LPS challenge. **(D)** Comparison of body temperature between LPS-treated piglets following 4 h (FO group) or 2 h (BT group) of LPS challenge. ^**^p < 0.01.

Table 3 shows the effect of maternal consumption of BT or FO on body temperature of piglets challenged with intraperitoneal injection of *E. coli* LPS. As expected, piglets experienced a rapid increase of body temperature within 4 h following LPS challenge. Despite the CON piglets also showed a gradual increase in body temperature which was finally kept below 39.5°C, the LPS-challenged piglets especially in the BT-LPS group reached more than 40°C within 2 h post LPS challenge. As a result, body temperature was significantly affected by the time (*p <* 0.0001) and LPS × Time interaction (*p <* 0.0001). Interestingly, the magnitudes of increase in body temperature approached to a marked (*p <* 0.01) difference between the BT-CON and BT-LPS piglets following 2 h of LPS challenge (Figure 1A), whereas the dramatic (*p <* 0.01) difference between the FO-CON and FO-LPS piglets was only observed at 4 h post LPS challenge (Figure 1B). Further comparison revealed that the body temperature of the BT-LPS piglets at 4 h was markedly higher (*p <* 0.01) than that of the FO-LPS piglets at 2 h (Figure 1C), whereas the body temperature of the FO-LPS piglets at 4 h only tended (*p <* 0.10) to be higher than that of the BT-LPS piglets at 2 h post LPS challenge (Figure 1D). These results indicated a delayed increase of body temperature in piglets from the FO-fed sows following LPS challenge. Overall, the body temperature averaged across the time points evaluated was about 0.2°C lower (*p <* 0.05) in the FO group than in the BT group.

**Table 3:** Effect of maternal diets on body temperature of piglets challenged with *E. coli* LPS.^a^

Body temperature, °C	BT		FO		*p*-value						
	CON	LPS	CON	LPS	Diet	LPS	Time	Diet × LPS	Diet × Time	LPS × Time	Diet × LPS × Time
0 h	38.73 ± 0.23	38.92 ± 0.24	38.52 ± 0.24	38.80 ± 0.34	^*^	^****^	^****^	NS	NS	^****^	NS
2 h	39.02 ± 0.35	40.08 ± 0.55	38.88 ± 0.26	39.67 ± 0.63							
4 h	39.47 ± 0.34	40.87 ± 0.55	39.42 ± 0.20	40.67 ± 0.36							

a BT, piglets from sows fed the diet supplemented with 4.6% of beef tallow; FO, piglets from sows fed the diet supplemented with 4.6% of fish oil; CON, piglets not challenged with LPS; LPS, piglets challenged with LPS. ^*^p < 0.05, and ^****^p < 0.0001; NS, *p* > 0.10.

### Aggravated inflammation and disturbed blood cell profile in piglets following LPS challenge

**Figure 2:**
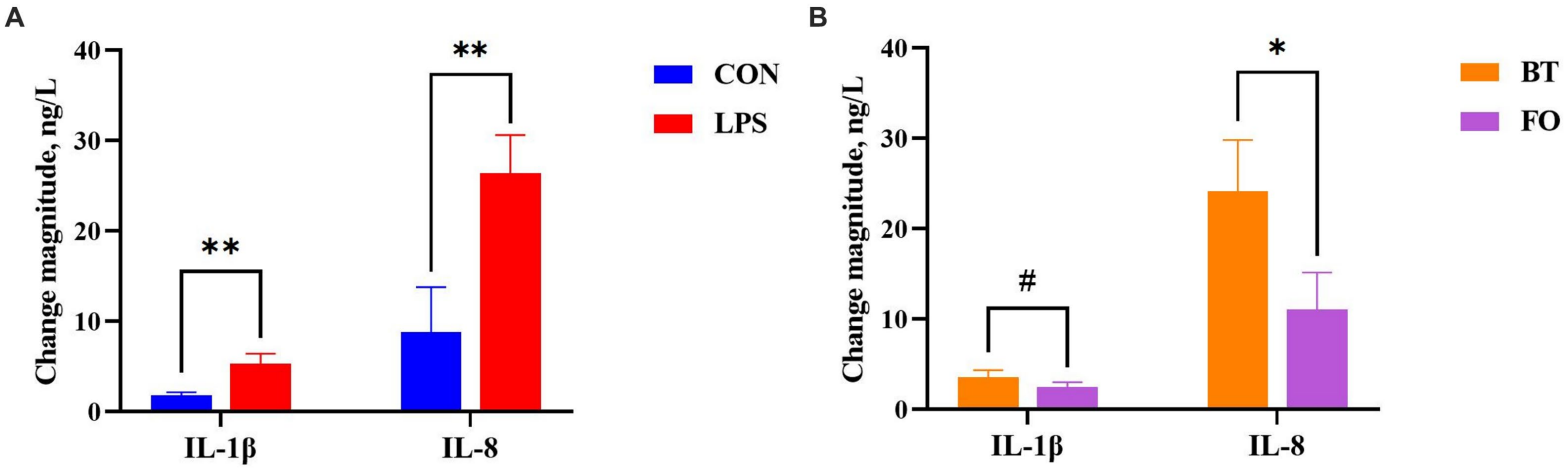
Comparison of the change magnitude of serum interleukin (IL)-1β and IL-8 levels, as evaluated by the difference between 4 h and 0 h post *E. coli* lipopolysaccharide (LPS) challenge, **(A)** between the CON and LPS groups and **(B)** between the beef tallow (BT) and fish oil (FO) groups. ^#^*p <* 0.10, ^*^p < 0.05, and ^**^p < 0.01.

As shown in Table 4, serum albumin levels were higher (*p <* 0.01) in the FO group than in the BT group, and were affected by the LPS × Time interaction (*p <* 0.01). Specifically, serum albumin levels were higher (*p <* 0.01) at 4 h than at 0 h following LPS challenge, and were higher (*p <* 0.01) in the LPS group than in the CON group at 4 h post LPS challenge. Serum IL-1β levels were affected by time (*p <* 0.0001) and the LPS × Time interaction (*p <* 0.05). Specifically, serum IL-1β levels were not different (*p* > 0.10) between the CON and LPS groups at 0 h, but were markedly enhanced (*p <* 0.0001) at 4 h post LPS challenge. Overall, serum IL-8 levels tended (*p <* 0.10) to be lower (77.71 vs. 96.41, ng/L) in the FO-fed than in the BT-fed sows, and tended (*p <* 0.10) to be higher (95.86 vs. 78.25, ng/L) at 4 h than at 0 h post LPS challenge. Further analysis indicated that the change magnitude of serum IL-1β and IL-8 levels, as evaluated by the difference between 4 h and 0 h, in the LPS-challenge group was about 3 times that in the CON group (Figure 2A). The FO group showed about 50% lower increase in both IL-1β (*p <* 0.10) and IL-8 (*p <* 0.05) levels at 4 h post LPS challenge compared with the BT group (Figure 2B). Table 5 shows effect of maternal consumption of BT or FO on blood cell counts of breast-fed piglets challenged with intraperitoneal injection of *E. coli* LPS. The blood cell counts of leukocyte (*p <* 0.001), neutrophil (*p <* 0.01) and lymphocyte (*p <* 0.01) were all markedly decreased at 4 h post LPS challenge. The significant effect of the LPS × Time interaction on leukocyte (*p <* 0.001), neutrophil (*p <* 0.01) and lymphocyte (*p <* 0.01) was manifested in that the dramatic decrease in these cells was mainly observed in the piglets challenged by LPS, but not observed in the BT-CON or FO-CON piglets that were not challenged with LPS. On the contrary, eosinophil percentage (*p <* 0.05), red blood cell counts (*p <* 0.01), hemoglobin concentration (*p <* 0.05), and hematocrit value were all increased following LPS challenge. Interestingly, piglets from the FO-fed sows tended (*p <* 0.10) to have decreased neutrophil counts and increased eosinophil percentage and RDW-SD, while had lower (*p <* 0.05) mean corpuscular hemoglobin concentration (MCHC) compared with the piglets from the BT-fed sows. The platelet hematocrit was lower (*p <* 0.01) at 4 h than at 0 h post LPS challenge, but there was a dramatic increase (*p <* 0.05) shortly after LPS challenge and then a marked decrease (*p <* 0.05) in the LPS-challenged piglets. Accordingly, a significant (*p <* 0.05) effect of the LPS × Time interaction on the platelet hematocrit was observed.

**Table 4:** Effect of maternal diets on serum albumin and cytokines levels of piglets challenged with *E. coli* LPS.^a^

Body temperature, °C	BT		FO		*p*-value						
	CON	LPS	CON	LPS	Diet	LPS	Time	Diet × LPS	Diet × Time	LPS × Time	Diet × LPS × Time
Albumin, g/L					^**^	NS	NS	NS	^#^	^**^	NS
0 h	22.53 ± 2.15	25.91 ± 2.83	28.46 ± 2.79	29.51 ± 3.33							
4 h	26.18 ± 2.44	22.83 ± 2.87	28.52 ± 2.52	23.28 ± 4.04							
IL-1β, ng/L					NS	NS	^****^	NS	NS	^*^	NS
0 h	16.70 ± 2.44	14.39 ± 2.02	16.00 ± 3.14	16.33 ± 1.45							
4 h	18.80 ± 2.83	21.58 ± 2.61	17.47 ± 2.42	19.77 ± 2.22							
IL-8, ng/L					^#^	NS	^#^	NS	NS	NS	NS
0 h	86.15 ± 18.45	82.53 ± 45.40	78.18 ± 28.49	66.15 ± 26.48							
4 h	97.50 ± 21.90	119.45 ± 38.21	84.41 ± 33.47	82.08 ± 30.37							

a BT, piglets from sows fed the diet supplemented with 4.6% of beef tallow; FO, piglets from sows fed the diet supplemented with 4.6% of fish oil; CON, piglets not challenged with LPS; LPS, piglets challenged with LPS. ^*^p < 0.05, ^**^p < 0.01, ^****^p < 0.0001, and ^#^*p <* 0.10; NS, *p* > 0.10.

**Table 5:** Effect of maternal diets on blood cell counts of piglets challenged with *E. coli* LPS.^a^

Body temperature, °C	BT		FO		*p*-value						
	CON	LPS	CON	LPS	Diet	LPS	Time	Diet × LPS	Diet × Time	LPS × Time	Diet × LPS × Time
Leukocyte, 10^9^/L					NS	^***^	^**^	NS	NS	^***^	NS
0 h	12.23 ± 5.74	12.03 ± 2.73	11.43 ± 3.63	12.47 ± 4.20							
4 h	15.86 ± 8.74	2.52 ± 2.69	9.47 ± 3.57	1.98 ± 1.08							
Neutrophil, 10^9^/L					^#^	^**^	^#^	NS	NS	^**^	NS
0 h	6.14 ± 4.78	5.55 ± 1.53	5.08 ± 2.59	5.12 ± 1.91							
4 h	8.95 ± 6.39	1.41 ± 2.03	4.20 ± 1.70	0.85 ± 0.67							
Lymphocyte, 10^9^/L					NS	^**^	^****^	NS	NS	^****^	NS
0 h	5.45 ± 3.01	5.91 ± 1.77	5.85 ± 1.32	6.78 ± 2.32							
4 h	6.23 ± 3.45	0.94 ± 0.48	4.92 ± 2.11	1.01 ± 0.41							
Monocyte, 10^9^/L					NS	^#^	^*^	NS	NS	^#^	NS
0 h	0.56 ± 0.50	0.47 ± 0.22	0.38 ± 0.22	0.46 ± 0.13							
4 h	0.60 ± 0.52	0.14 ± 0.20	0.28 ± 0.09	0.09 ± 0.06							
Eosinophil, 10^9^/L					NS	NS	^**^	NS	NS	NS	NS
0 h	0.08 ± 0.05	0.10 ± 0.03	0.12 ± 0.06	0.11 ± 0.08							
4 h	0.07 ± 0.05	0.03 ± 0.05	0.07 ± 0.05	0.03 ± 0.02							
Neutrophil, %					NS	NS	NS	NS	NS	NS	NS
0 h	44.55 ± 20.87	46.25 ± 7.26	41.93 ± 13.39	40.68 ± 5.05							
4 h	54.28 ± 11.90	43.12 ± 22.72	44.62 ± 9.11	37.13 ± 17.06							
Lymphocyte, %					NS	NS	NS	NS	NS	NS	NS
0 h	50.62 ± 22.52	49.03 ± 8.26	54.00 ± 14.44	54.58 ± 5.92							
4 h	41.70 ± 11.32	50.73 ± 23.05	51.60 ± 9.52	56.40 ± 16.29							
Monocyte, %					NS	NS	NS	NS	NS	NS	NS
0 h	4.13 ± 2.07	3.95 ± 1.38	3.08 ± 1.27	3.93 ± 1.54							
4 h	3.57 ± 1.26	4.88 ± 2.39	3.05 ± 0.90	4.33 ± 2.30							
Eosinophil, %					^#^	^*^	NS	NS	NS	^**^	NS
0 h	0.70 ± 0.29	0.77 ± 0.15	0.98 ± 0.29	0.80 ± 0.35							
4 h	0.45 ± 0.24	1.25 ± 1.06	0.73 ± 0.37	1.85 ± 1.11							
RBC, 12^9^/L					NS	^**^	^**^	NS	NS	NS	NS
0 h	4.87 ± 1.48	5.99 ± 0.38	5.93 ± 0.67	5.81 ± 0.71							
4 h	6.08 ± 0.96	7.58 ± 2.26	5.79 ± 0.61	7.60 ± 1.27							
Hemoglobin, g/L					NS	^*^	^**^	NS	NS	NS	NS
0 h	86.83 ± 30.02	107.83 ± 14.82	107.17 ± 12.42	100.17 ± 9.45							
4 h	107.17 ± 25.33	133.67 ± 37.75	104.67 ± 11.08	131.00 ± 21.40							
Hematocrit value, %					NS	^*^	^**^	NS	NS	NS	NS
0 h	27.68 ± 10.09	34.87 ± 4.86	34.87 ± 3.43	32.42 ± 3.44							
4 h	35.17 ± 8.55	42.77 ± 12.23	34.43 ± 3.31	42.93 ± 6.57							
MCV, fL					NS	NS	NS	NS	NS	NS	NS
0 h	56.08 ± 8.02	57.98 ± 5.10	59.17 ± 5.97	55.93 ± 2.62							
4 h	57.27 ± 8.30	56.52 ± 4.12	59.78 ± 5.90	56.65 ± 2.89							
MCH, pg					NS	NS	NS	NS	NS	NS	NS
0 h	17.75 ± 2.38	17.95 ± 1.74	18.17 ± 1.76	17.25 ± 0.82							
4 h	17.48 ± 2.44	17.72 ± 1.39	18.18 ± 1.91	17.20 ± 0.69							
MCHC, g/L					^*^	NS	^#^	NS	NS	NS	^#^
0 h	316.83 ± 14.76	309.17 ± 7.36	307.33 ± 6.35	309.00 ± 8.65							
4 h	305.67 ± 4.13	313.17 ± 3.31	304.00 ± 3.46	304.00 ± 7.48							
RDW-CV, %					NS	NS	NS	NS	NS	NS	NS
0 h	36.57 ± 1.75	30.48 ± 7.87	34.58 ± 5.64	36.42 ± 2.40							
4 h	36.85 ± 13.90	32.77 ± 5.07	34.98 ± 5.60	37.08 ± 2.17							
RDW-SD, fL					^#^	NS	NS	NS	NS	NS	NS
0 h	73.82 ± 17.66	65.35 ± 12.30	76.48 ± 9.89	76.98 ± 4.63							
4 h	75.67 ± 18.19	69.40 ± 6.43	77.75 ± 9.36	79.62 ± 4.99							
Platelet, 10^9^/L					NS	NS	^**^	NS	NS	^*^	NS
0 h	167.83 ± 68.00	259.33 ± 100.40	180.33 ± 60.32	262.00 ± 152.18							
4 h	157.50 ± 81.28	123.00 ± 76.09	156.33 ± 94.31	98.17 ± 33.15							
MPV, fL					NS	NS	^*^	NS	NS	NS	NS
0 h	8.78 ± 1.54	8.73 ± 1.17	8.63 ± 1.13	8.07 ± 0.92							
4 h	8.35 ± 1.08	7.95 ± 0.77	8.03 ± 0.90	7.35 ± 0.96							
PDW, %					NS	NS	NS	^*^	NS	NS	NS
0 h	15.37 ± 0.72	15.97 ± 0.82	15.72 ± 0.41	15.48 ± 0.39							
4 h	15.28 ± 0.94	15.38 ± 0.57	15.65 ± 0.46	15.27 ± 0.39							
PCT, %					NS	NS	^**^	NS	NS	^*^	NS
0 h	0.15 ± 0.07	0.23 ± 0.09	0.16 ± 0.06	0.22 ± 0.15							
4 h	0.13 ± 0.06	0.10 ± 0.07	0.13 ± 0.08	0.07 ± 0.04							

a BT, piglets from sows fed the diet supplemented with 4.6% of beef tallow; FO, piglets from sows fed the diet supplemented with 4.6% of fish oil; CON, piglets not challenged with LPS; LPS, piglets challenged with LPS. RBC, red blood cell; MCV, mean corpuscular volume; MCH, mean corpuscular hemoglobin; MCHC, mean corpuscular hemoglobin concentration; RDW-CV, red cell distribution width coefficient of variance; RDW-SD, red cell distribution width standard deviation; MPV, mean platelet volume; PDW, platelet distribution width; PCT, platelet hematocrit. ^*^p < 0.05, ^**^p < 0.01, ^***^p < 0.001, ^****^p < 0.0001, and ^#^*p <* 0.10; NS, *p* > 0.10.

### Atrophied villi and reshaped fatty acids in piglet intestine following LPS challenge

**Figure 3:**
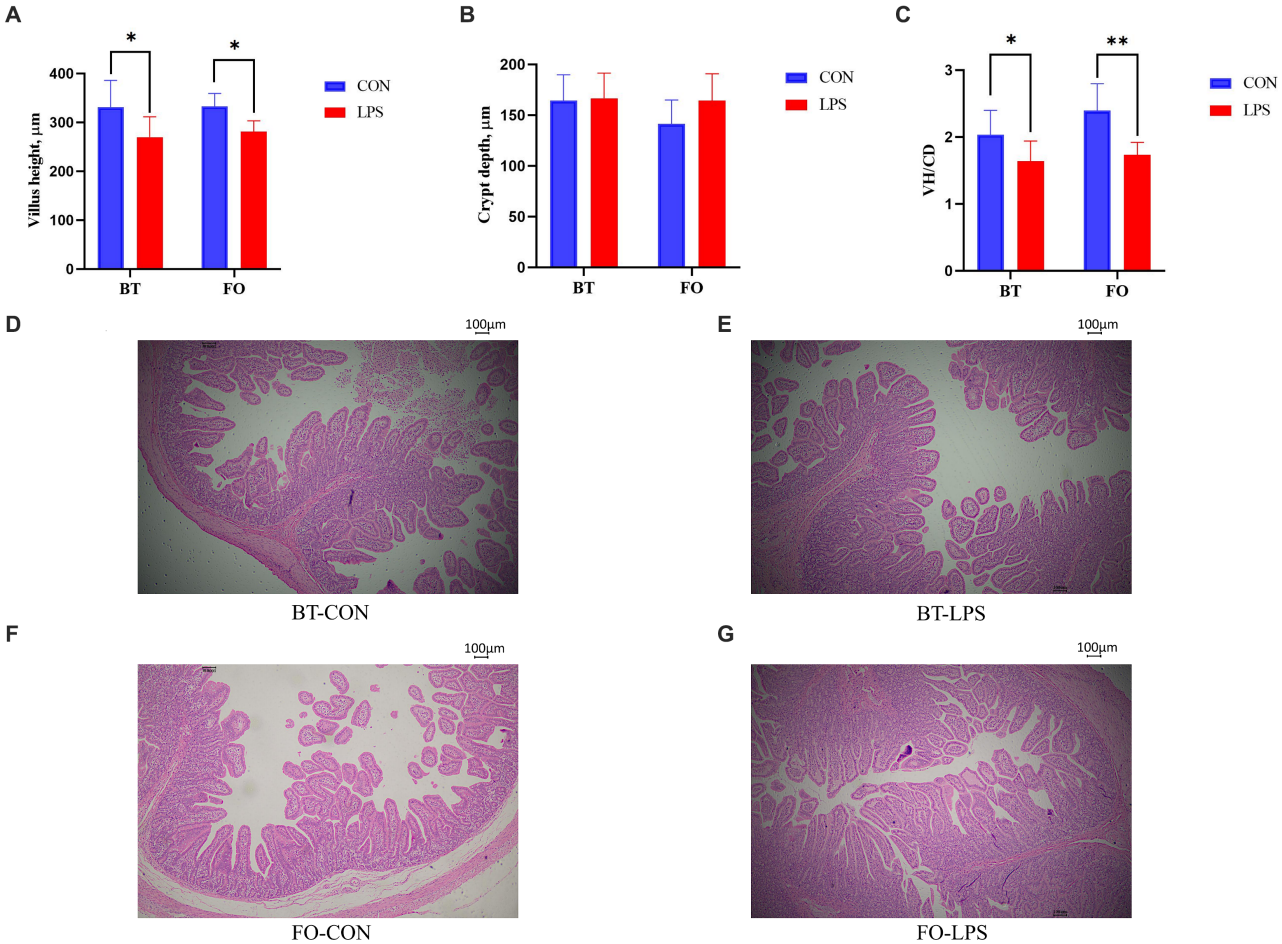
Effect of maternal consumption of beef tallow (BT) or fish oil (FO) on the intestinal morphology of piglets challenged with intraperitoneal injection of *E. coli* lipopolysaccharide (LPS). Comparison of **(A)** villus height (VH), **(B)** crypt depth (CD) and **(C)** ratios of VH to CD (VH/CD) of jejunum of piglets following 4 h of LPS challenge between the CON and LPS-treated piglets. The representative figures (40×) of **(D)** BT-CON, **(E)** BT-LPS, **(F)** FO-CON and **(G)** FO-LPS treatments. ^*^p < 0.05 and ^**^p < 0.01.

**Figure 4:**
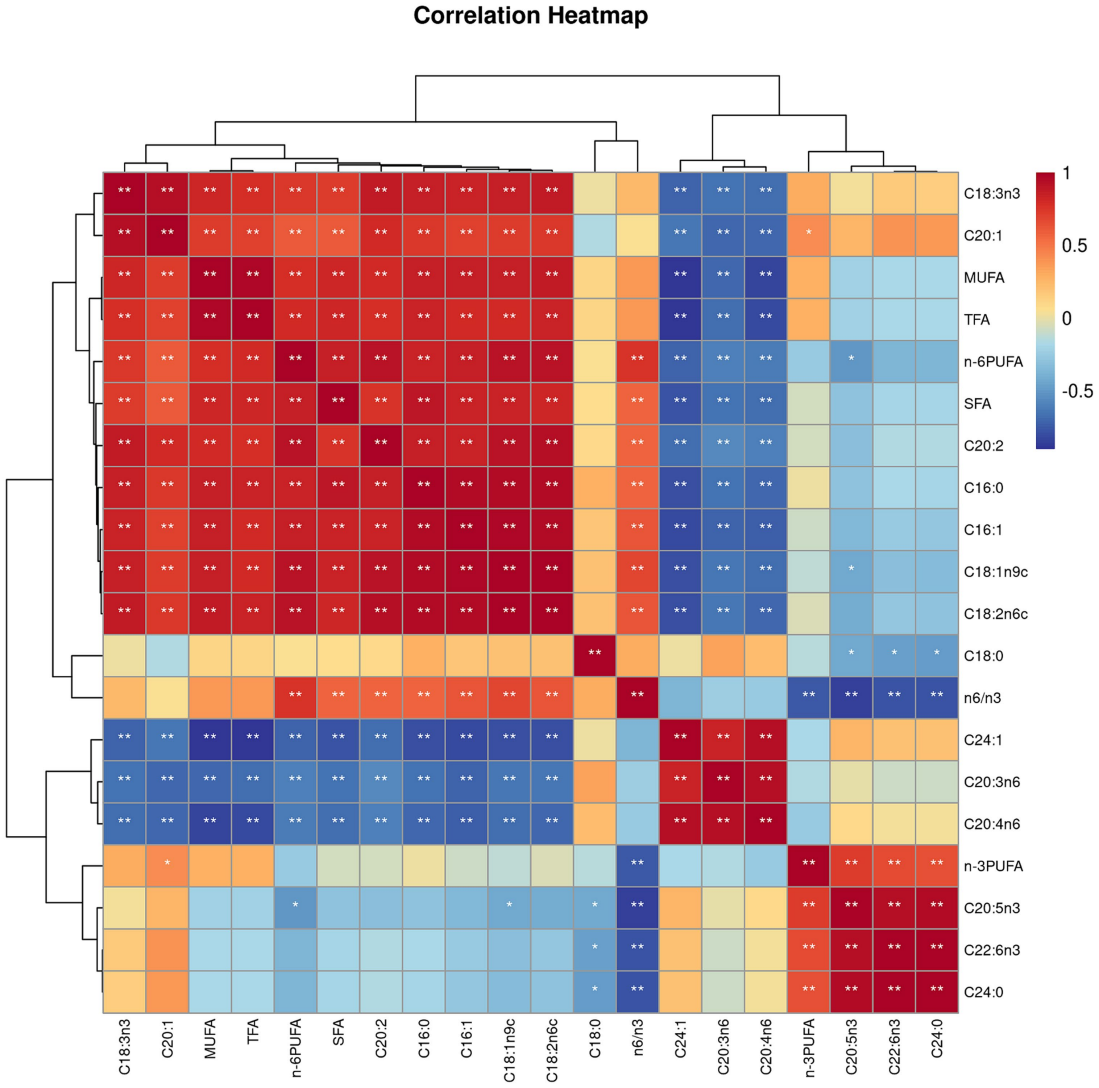
Correlation heatmap of fatty acids detected in milk of sows fed beef tallow (BT) or fish oil (FO) diet and in ileum tissues of breast-fed piglets not challenged with intraperitoneal injection of *E. coli* lipopolysaccharide (LPS). ^*^p < 0.05 and ^**^p < 0.01.

**Figure 5:**
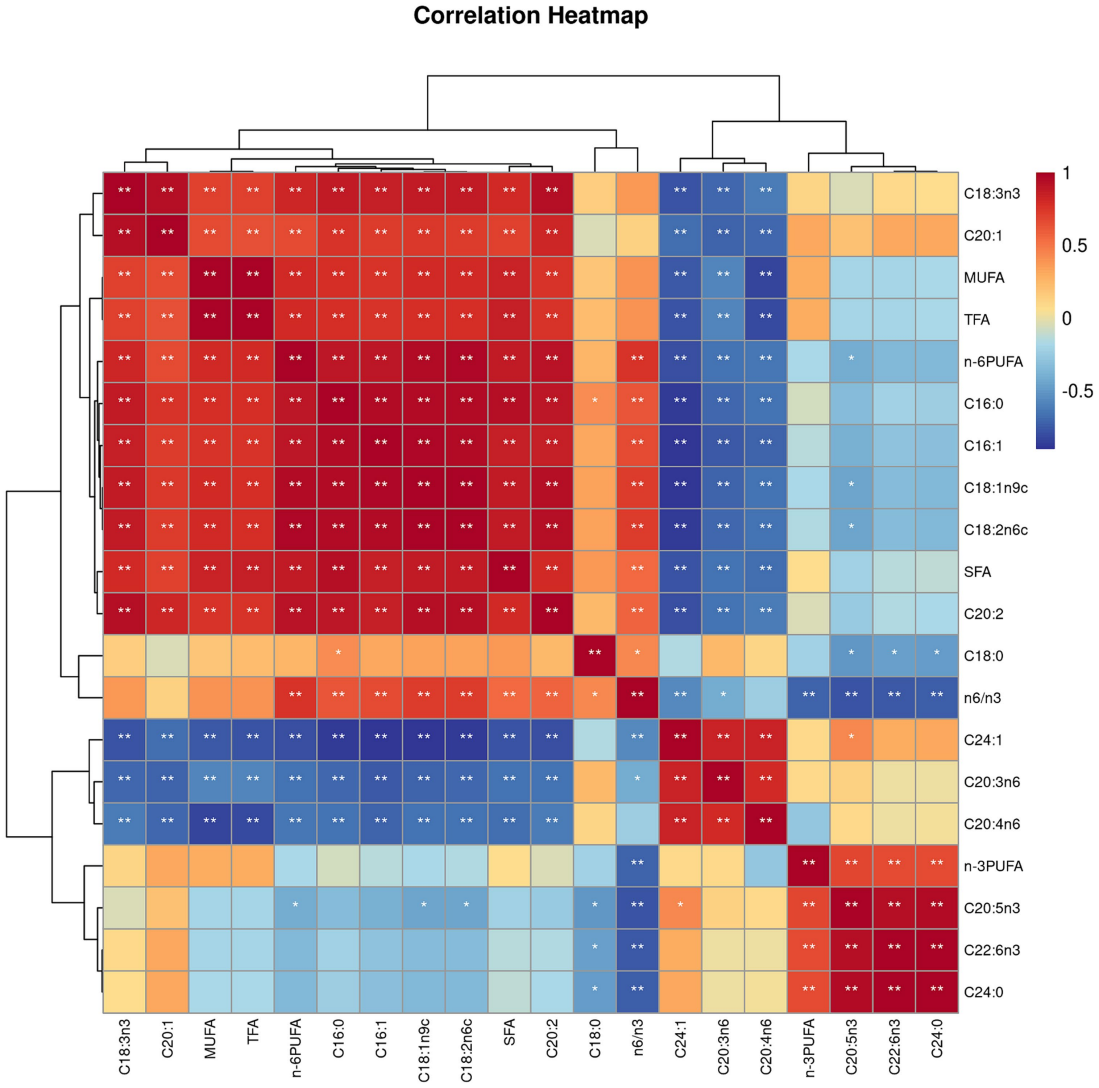
Correlation heatmap of fatty acids detected in milk of sows fed beef tallow (BT) or fish oil (FO) diet and in ileum tissues of breast-fed piglets challenged with intraperitoneal injection of *E. coli* lipopolysaccharide (LPS). ^*^p < 0.05 and ^**^p < 0.01.

Both villus height and the ratios of villus height to crypt depth (VH/CD) of piglets were decreased (*p <* 0.05 or *p <* 0.01) following 4 h of LPS challenge regardless of dietary treatments (Figure 3). However, piglets from the FO-fed sows appeared to have relatively higher (*p* = 0.10) VH/CD than those from the BT-fed sows (Figure 3). Table 6 shows effect of maternal consumption of BT or FO on intestinal fatty acid profile of piglets challenged with intraperitoneal injection of *E. coli* LPS. A total of 14 fatty acid species were detected in the ileum tissue. Compared with piglets from the BT-fed sows, piglets from the FO-fed sows had lower intestinal content of C16:1 (*p <* 0.05), C18:1n9c (*p <* 0.001), C18:2n6c (*p <* 0.05), C20:2 (*p <* 0.01), and C20:4n6 (*p <* 0.0001), but much higher (*p <* 0.0001) content of C20:5n3, C24:0, and C22:6n3, which were 2–4 times the content of piglets from the BT-fed sows. Intestinal content of C16:0 tended (*p <* 0.10) to be lower, while C20:1 tended (*p <* 0.10) to be higher in piglets from the FO-fed sows than in piglets from the BT-fed sows. LPS challenge resulted in decreased (*p <* 0.05) intestinal C20:1 and C20:5n3 content. Interestingly, intestinal C20:3n6 and C24:1 content was significantly affected (*p <* 0.05) by the Diet × LPS interaction. Further comparison revealed that LPS challenge induced decrease (*p <* 0.05) in intestinal C20:3n6 and C24:1 content occurred in the BT-LPS piglets rather than in the FO-LPS piglets when compared with their respective CON piglets. Further analysis indicated that intestinal fatty acid composition and levels were highly correlated with milk fatty acids constituents either in the CON piglets (Figure 4) or in the LPS-treated piglets (Figure 5). Long-chain fatty acids, especially n-3 PUFA, play a pivotal role in determining the trend of changes in the strength of correlation in the cluster analysis. Remarkably, as shown in the correlation heatmap, there are differences in the types of fatty acids arranged from right to left in the 10th to 15th positions which were C18:2n6c, C18:1n9c, C16:1, C16:0, C20:2, and SFA in the CON piglets (Figure 4), in contrast to C20:2, SFA, C18:2n6c, C18:1n9c, C16:1, and C16:0 in the LPS-treated piglets (Figure 5). These results indicated the reshaping of ileum fatty acid profile of breast-fed piglets following maternal consumption of varied types of fatty acids or following LPS challenge.

**Table 6:** Effect of maternal diets on ileum fatty acid profile of piglets challenged with *E. coli* LPS.^a^

	BT		FO		*p*-value		
	CON	LPS	CON	LPS	Diet	LPS	Diet × LPS
**SFA**							
C16:0	5.22 ± 1.27	5.08 ± 0.96	4.81 ± 1.35	3.79 ± 1.06	^#^	NS	NS
C18:0	2.85 ± 0.40	2.72 ± 0.61	2.90 ± 0.57	2.55 ± 0.56	NS	NS	NS
C24:0	0.11 ± 0.03	0.08 ± 0.01	0.30 ± 0.05	0.32 ± 0.03	^****^	NS	^#^
∑SFA	8.18 ± 1.53	7.88 ± 1.56	8.00 ± 1.93	6.66 ± 1.60	NS	NS	NS
**MUFA**							
C16:1	0.84 ± 0.34	0.83 ± 0.21	0.70 ± 0.31	0.44 ± 0.26	^*^	NS	NS
C18:1n9c	4.65 ± 1.38	4.47 ± 1.24	3.03 ± 1.10	2.18 ± 0.87	^***^	NS	NS
C20:1	0.07 ± 0.01	0.06 ± 0.02	0.10 ± 0.03	0.06 ± 0.03	^#^	^*^	NS
C24:1	0.17 ± 0.04	0.14 ± 0.01	0.14 ± 0.01	0.15 ± 0.02	NS	NS	^*^
∑MUFA	5.73 ± 1.69	5.49 ± 1.43	3.97 ± 1.44	2.83 ± 1.14	^**^	NS	NS
**n-6 PUFA**							
C18:2n6c	2.04 ± 0.36	1.93 ± 0.37	1.74 ± 0.66	1.28 ± 0.32	^*^	NS	NS
C20:2	0.06 ± 0.01	0.05 ± 0.01	0.05 ± 0.01	0.04 ± 0.01	^**^	NS	NS
C20:3n6	0.07 ± 0.01	0.05 ± 0.01	0.06 ± 0.01	0.06 ± 0.01	NS	NS	^*^
C20:4n6	1.13 ± 0.23	0.98 ± 0.11	0.59 ± 0.05	0.63 ± 0.09	^****^	NS	NS
∑n-6 PUFA	3.29 ± 0.32	3.01 ± 0.45	2.43 ± 0.70	2.01 ± 0.29	^****^	^#^	NS
**n-3 PUFA**							
C18:3n3	0.05 ± 0.01	0.05 ± 0.01	0.06 ± 0.03	0.03 ± 0.03	NS	NS	NS
C20:5n3	0.08 ± 0.01	0.06 ± 0.00	0.17 ± 0.02	0.15 ± 0.02	^****^	^*^	NS
C22:6n3	0.16 ± 0.04	0.13 ± 0.01	0.64 ± 0.14	0.56 ± 0.10	^****^	NS	NS
∑n-3 PUFA	0.28 ± 0.04	0.24 ± 0.02	0.86 ± 0.19	0.74 ± 0.12	^****^	^#^	NS
Total fatty acids	17.49 ± 3.41	16.62 ± 3.41	15.26 ± 4.20	12.24 ± 3.10	^*^	NS	NS

a BT, piglets from sows fed the diet supplemented with 4.6% of beef tallow; FO, piglets from sows fed the diet supplemented with 4.6% of fish oil; CON, piglets not challenged with LPS; LPS, piglets challenged with LPS. ^*^p < 0.05, ^**^p < 0.01, ^***^p < 0.001, ^****^p < 0.0001, and ^#^*p <* 0.10; NS, *p* > 0.10.

## Discussion

Maternal consumption of FO enhancing the weaning survival rate of breast-fed piglets have been indicated in our previous study (35). And it was proposed that FO consumption by sows might benefit the piglets via increasing n-3 PUFA (particularly C20:5n3 and C22:6n3) availability and immunoglobulins (IgG and IgM) secretion (35). However, it remains unclear whether the increase in n-3 PUFA availability could enhance the ability of piglets to resist pathogen invasion. Pathogenic *E. coli* has been identified to be one of the main etiologic agents causing yellow or white dysentery in nursery pigs (27), and moreover, the inflammatory diseases caused by *E. coli* are mainly related to the presence of LPS in the outer cell membrane (4–6). Thus, in present study LPS was used as the stressor to create an immune stress model to test the potential protection effect of maternal n-3 PUFA consumption on breast-fed piglets. Consistent with previous studies, LPS challenge resulted in a dramatic increase of body temperature (31–33, 38). Blood cells also presented a quick response to LPS challenge (40–42). The increased body temperature combined with the decrease in blood leukocyte, lymphocyte and neutrophil counts and the increase in eosinophil percentage, red blood cell counts, hemoglobin concentration and hematocrit value following LPS challenge provided strong evidence for the establishment of LPS-induced immune stress model.

One important finding in present study is the delayed increase of body temperature of LPS-challenged piglets from the FO-fed sows. Firstly, in contrast to a quick increase of body temperature following LPS challenge in piglets from the BT-fed sows, the marked increase of body temperature in piglets from the FO-fed sows was only observed at 4 h post LPS challenge. This result indicated the significant effect of nutritional status of nursery pigs on their response to LPS challenge. Consistent with this notion, the body temperature of LPS-challenged weaned piglets was also shown to be affected by their diets (38, 43–46). However, different to the current study, some of weaned piglets showed a decrease of body temperature at 2 h post LPS challenge (38), which might be explained by the difference in the age of pigs subjected to LPS challenge and the difference in nutritional source. On one hand, the increase of body temperature indicated the enhanced energy metabolism to meet the energy requirement of the organism to resist pathogen invasion (47–53). In support of this viewpoint, the consumption of butyrate, a direct fuel of enterocytes to obtain energy (54), has been shown to prevent the decrease of body temperature following LPS challenge (38). On the other hand, the decrease of body temperature suggested the disturbed energy metabolism (38), which might be caused by the insufficient supply of oxygen that was needed for oxidative decomposition of energy substances. As observed in present study, the increase in red blood cell counts and hemoglobin concentration provided evidence for the increased requirement of oxygen supply under LPS challenge. The observation that the body temperature averaged across the time points evaluated was about 0.2°C higher in the BT group than in the FO group implied the increased energy expenditure in the piglets from the BT-fed sows. The relatively higher mean corpuscular hemoglobin concentration (MCHC) in piglets from the BT-fed sows might lay a physiological basis for their increased energy expenditure.

Another important finding in present study is the reshaping of intestinal fatty acid profile of the FO group piglets following LPS challenge. It has been well established that milk fatty acid composition could be regulated by dietary fat types. Earlier studies in sows indicated that when fish oil rich in n-3 PUFA (mainly EPA and DHA), was added at 7% of the diet, the content of total n-3 PUFA in milk was increased by about 8 times, and the content of DHA was increased by about 35 times, compared with the same proportion (7%) of lard supplementation (55). Further studies found that when fat sources were supplemented at 3.8–3.9% of diets, DHA contents in sow milk and piglet serum were increased by about 30 and 3 times, respectively, in the fish oil group compared with the palm oil group (35). In present study, when fat sources (FO vs. BT) were added at 4.6% of diets, the total n-3 PUFA in the milk of the FO-fed sows were about 5 times the content of the BT-fed sows, whereas the total n-3 PUFA in the ileum of piglets from the FO-fed sows were just 3 times the content in the ileum of piglets from the BT-fed sows. Noting that except for C18:3n3 (0.32 g/kg milk) and minor amount of C20:3n3 (0.05 g/kg milk), EPA and DHA were not detected in the milk of the BT-fed sows, but they were detected in piglet ileum to be 0.08 and 0.16 g/kg tissue, respectively. These results indicated the synthesis of long-chain n-3 PUFA, EPA and DHA, in breast-fed piglets by using milk derived C18:3n3 and/or C20:3n3. Similarly, the ratios of C20:3n6 and C20:4n6 to C18:2n6c being higher in the ileum tissues than in the sow milk indicated the long-chain n-6 PUFA synthesis in piglets. This could explain the highly negative correlation of ileum n-6 PUFA with milk C20:3n6 and C20:4n6, which is in contrast to the highly positive correlation of ileum n-6 PUFA with milk C18:2n6c.

The enzymes including FADS1 and FADS2 that appear responsible for all n-3 and n-6 PUFA desaturation have been identified in mammals (36), which might provide a biochemical basis for the reshaping of ileum fatty acids following LPS challenge. Specifically, the ileum DGLA (C20:3n6) and EPA (C20:5n3) contents were markedly decreased following LPS challenge in the piglets from the BT-fed sows, suggesting the increased demand of DGLA and EPA by enterocytes subjected to LPS challenge. Given evidence that there is a beneficial effect of DGLA on the level of brain-derived neurotrophic factor (BDNF) which can interact with inflammation as the risk factor in the cardiovascular disorders (56), including stroke, the relatively stable DGLA level in the FO piglets might be beneficial for piglets to survive epically when they were facing the increased body temperature under LPS challenge. The differences in the types of fatty acids arranged from right to left in the 10th to 15th positions in the correlation heatmap further indicated the difference of fatty acid metabolism when the organism is subjected to pathogens invasion or not. It has been demonstrated that n-3 PUFA, via *in vitro* stimulation or via dietary supplementation, effectively incorporate into the cellular membrane of all the immune cells (57–59). n-3 PUFA and their metabolites are natural ligands for PPAR-γ (60), and PPAR-γ is a transcription factor that acts in an anti-inflammatory manner (61). The anti-inflammatory effect of n-3 PUFA via the activation of PPAR-γ and inhibition of NFκB has been confirmed by a series of studies (19, 22–24, 26). In present study, the lower increase in proinflammatory cytokines (IL-1β and IL-8) levels, the higher serum albumin concentrations, enhanced ratios of villus height to crypt depth, and the delayed increase of body temperature following LPS challenge suggested the beneficial effect of maternal n-3 PUFA consumption on their progeny. Thus, there is reason to believe that the reshaped intestinal fatty acid profile has contributed to the improvement of the ability of piglets to resist LPS invasion.

## Conclusion

In summary, this study indicated that maternal consumption of fish oil protected breast-fed piglets against *E. coli* lipopolysaccharide-induced injury through reshaping of intestinal fatty acids profile, which sheds new light on the development of nutritional strategies to enhance the ability of young pigs to resist *E. coli* invasion.

## Data availability statement

The original contributions presented in the study are included in the article/supplementary material, further inquiries can be directed to the corresponding author.

## Ethics statement

The animal study was approved by Animal Care and Use Committee of the Animal Nutrition Institute (2022092114008), Sichuan Agricultural University. The study was conducted in accordance with the local legislation and institutional requirements.

## Author contributions

BF: Writing – review & editing, Writing – original draft, Methodology, Investigation, Formal analysis, Data curation. LZ: Writing – review & editing, Methodology, Investigation, Formal analysis, Data curation. BH: Writing – review & editing, Methodology, Investigation. FC: Writing – review & editing, Methodology. PY: Writing – review & editing, Methodology, Investigation. SL: Writing – review & editing, Methodology, Investigation. AW: Writing – review & editing, Methodology, Investigation. YZ: Writing – review & editing, Validation, Supervision, Resources, Funding acquisition, Conceptualization.
